# Effects of electronic cigarette e-liquid flavouring on cigarette craving

**DOI:** 10.1136/tobaccocontrol-2021-056769

**Published:** 2021-11-17

**Authors:** Maddy L Dyer, Jasmine N Khouja, Abigail R Jackson, Michelle A Havill, Martin J Dockrell, Marcus R Munafo, Angela S Attwood

**Affiliations:** 1 School of Psychological Science, University of Bristol, Bristol, UK; 2 Medical Research Council Integrative Epidemiology Unit, University of Bristol, Bristol, UK; 3 Cardiff School of Sport and Health Sciences, Cardiff Metropolitan University, Cardiff, UK; 4 Department of Health Improvement, Public Health England, London, UK; 5 National Institute for Health Research Bristol Biomedical Research Centre, University Hospitals Bristol NHS Foundation Trust, Bristol, UK

**Keywords:** electronic nicotine delivery devices, nicotine, public policy

## Abstract

**Background:**

E-liquid flavour restrictions may discourage electronic cigarette (e-cigarette) uptake among youth. However, possible unintended consequences may include reduced appeal and effectiveness of e-cigarettes for smoking cessation. Non-tobacco flavours appear to be important for smoking cessation, but how and why are currently unclear.

**Methods:**

We conducted an experimental study in a UK sample of adult daily smokers using an independent groups design (N=84). Participants were randomised to use an e-cigarette with nicotine-containing fruit/sweet-flavoured e-liquid (blackcurrant, strawberry, vanilla, caramel) or unflavoured e-liquid for 1 week. The primary outcomes were average, peak and cue-elicited cigarette craving (the latter was assessed using a cue exposure task). The secondary outcomes were smoking lapse occurrence, enjoyment of the e-cigarette, ease of transitioning from smoking to using an e-cigarette, intentions to continue using an e-cigarette, intentions and motivation to quit smoking, return to smoking, and continuation of e-cigarette use.

**Results:**

E-liquid flavouring did not appear to have an effect on average cigarette craving (*b* 0.18, 95% CI −0.44 to 0.79, p=0.57), peak cigarette craving (*b* −0.12, 95% CI −0.59 to 0.35, p=0.62) or cue-elicited cigarette craving (*b* −0.21, 95% CI −3.86 to 3.43, p=0.91). We did not find evidence of a difference in secondary outcomes.

**Conclusions:**

We did not find evidence to suggest that nicotine-containing fruit/sweet-flavoured and unflavoured e-liquids have different effects on cigarette cravings after 1 week of use. Further research is needed to establish if differences emerge over longer periods of exposure and extend to smoking cessation outcomes.

## Introduction

There are thousands of electronic cigarette (e-cigarette) e-liquid flavours available, including tobacco, menthol/mint, fruit, sweet/dessert and drink flavours.^1^ Among adult e-cigarette users in Great Britain, the main reasons reported for use are to aid smoking cessation (30%) and smoking reduction (11%) and to prevent relapse (20%).^2^ Among ever users aged 11–18 years old, the reasons are experimentation (50%), flavour liking (13%) (6% among never smokers) and conformity (13%).^3^ The availability of flavoured e-liquids is controversial; while flavoured e-liquids may aid smoking cessation,^4^ they may also encourage e-cigarette use among youth. Therefore, internationally there is debate among policy makers regarding the possible benefits and costs of e-liquid flavour restrictions.

Globally, 11 countries and 28 European Union Member States have implemented policies restricting the availability or marketing of flavoured nicotine-containing products,^5^ including 13 countries regulating e-cigarette ingredients and flavours,^6^ citing public health protection as the rationale. For example, Canada has prohibited flavour descriptors and imagery on e-cigarette products,^5^ whereas the USA has banned the sale of flavoured cartridge-based e-cigarette products (except tobacco and menthol).^7 8^ This US regulation was imposed following lung injuries and fatalities mostly associated with vaping products containing tetrahydrocannabinol oil and vitamin E acetate,^9^ and concerns about e-cigarette uptake among youth.^7^ Indeed, flavour variety is one of the primary appeals of e-cigarettes among youth,^8^ and flavours promote initiation and higher frequency of use among young adult e-cigarette users.^10^ Fruit flavours are the most popular flavours among youth e-cigarette users^3 11^ and are perceived as less harmful than tobacco flavours among adolescent non-users.^12^ Furthermore, e-cigarette use in adolescence is associated with subsequent smoking.^13^ However, it is unclear whether this reflects a causal pathway; findings from observational studies may be explained by shared common causes of e-cigarette use and smoking (eg, risk-taking propensity), rather than a gateway effect.^14 15^

Others have argued that e-liquid flavour restrictions may have unintended consequences, for example by reducing the appeal and effectiveness of e-cigarettes for smoking reduction and cessation.^16 17^ In an international survey, adult former smokers report that flavour variability was ‘very important’ when attempting to quit smoking and that restricting this would reduce enjoyment and increase cigarette craving.^18^ A report from Great Britain indicated that if flavours were no longer available, 25% of adult e-cigarette users would still try to get them and 10% would make their own,^2^ which indicate possible use of black markets and unregulated products. However, 18% said they would use unflavoured e-liquids/cartridges. Nevertheless, approximately 20% said that they would smoke more or return to smoking and approximately 8% would stop using e-cigarettes.^2 17^ This is problematic, as current evidence suggests they are considerably less harmful than cigarettes.^17 19^ Furthermore, the most comprehensive living systematic review to date suggests that, among adult smokers who attempt to stop smoking, smoking quit rates are higher in those randomised to nicotine-containing e-cigarettes versus nicotine replacement therapy or behavioural support only/no support.^20^

Researchers have investigated the rewarding effects of e-liquid flavours. One survey found that adult e-cigarette users who use candy or fruit (vs tobacco) flavours reported more satisfaction from using e-cigarettes (vs smoking) and more enjoyment of vaping.^21^ Experimental research found that cherry and menthol (vs unflavoured and tobacco) flavoured e-liquids were more pleasant, among adult cigarette smokers naïve to e-cigarettes,^22^ and apple and chocolate flavoured e-liquids were more rewarding than unflavoured e-liquids among young adult smokers.^23^

E-cigarettes attenuate cravings for cigarettes,^24–26^ which is an important motivation for use among smokers.^27^ Higher nicotine concentrations and more intensive use are associated with reduced cigarette craving among recent quitters.^28^ However, there is limited experimental evidence regarding the role of e-liquid flavouring in smoking cessation, including effects on cigarette craving. One randomised trial found adult daily smokers had lower smoking urges when using cherry flavoured e-liquids compared with tobacco, menthol, espresso and vanilla flavours (24 mg/mL).^29^ Another within-subjects experimental study found apple and apple/menthol flavoured nicotine-free e-liquids were associated with higher cigarette cravings than menthol flavoured e-liquid among young adult smokers.^30^ However, participants in the latter study were menthol-preferring smokers; therefore, menthol was likely a smoking cue and findings may not be generalisable to all smokers. Additionally, neither study had an unflavoured control condition, which is important because unflavoured e-liquids are exempt from some restrictions. As cigarette craving intensity predicts smoking relapse,^31^ determining the effect of e-liquid flavouring on cigarette craving is vital. Furthermore, it is important to examine cue-elicited cigarette craving, the desire to smoke following exposure to conditioned cues,^32^ as smoking behaviour can be promoted by conditioned stimuli associated with smoking.^33^

Our primary aim was to investigate the effects of using e-cigarettes with flavoured (ie, fruit/sweet) versus unflavoured (ie, no flavour) nicotine-containing e-liquid for 1 week on (1) general (average and peak) cigarette craving and (2) cue-elicited cigarette craving in response to smoking-related cues, among abstinent daily smokers. We used a cue exposure task for (1) as similar procedures with visual, auditory, olfactory and tactile smoking cues reliably increase cigarette craving.^34–36^ Although smokers are most interested in trying tobacco (30%) and menthol/mint (18%) flavours,^37^ we excluded these flavours because they are associated with cigarettes and are typically exempt from restrictions. Non-tobacco flavours may disrupt conditioned associations between smoking cues (eg, tobacco smell/taste) and the rewarding properties of nicotine, for example, by creating new flavour associations. We therefore hypothesised that participants randomised to use an e-cigarette with flavoured (vs unflavoured) e-liquid would report lower (1) general cigarette craving and (2) cue-elicited cigarette craving. We also explored the effect of e-cigarette flavouring on (1) smoking lapse occurrence, (2) enjoyment of the e-cigarette, (3) ease of transitioning from smoking to using an e-cigarette, (4) intentions to continue using an e-cigarette, (5) intentions to quit smoking, (6) motivation to quit smoking, and (7) return to smoking and (8) continuation of e-cigarette use. Finally, we explored the effects of e-liquid flavouring on food cravings, given the relationship between smoking and food intake.^38^

## Methods

### Design

We conducted an experimental study with an independent groups design. E-liquid flavouring (flavoured vs unflavoured) was assigned using a random number generator (http://www.randomizer.org) by ASA. ARJ and JNK enrolled participants and were blind until the point of allocation. We planned to conduct the study at the University of Bristol. However, due to the COVID-19 pandemic, all participants were tested remotely. The study protocol was preregistered (https://osf.io/jtgxc/).

### Participants

We recruited 84 participants via Facebook and Twitter (~55%), the Tobacco and Alcohol Research Group website and mailing lists (~20%), and word of mouth (~25%) from July to November 2020. Participants were eligible if they were ≥18 years old, residing in the UK, a daily smoker (≥5 cigarettes per day for ≥3 months), not currently attempting to quit smoking or using an e-cigarette (to exclude individuals currently altering their smoking behaviour), willing to replace all cigarettes with an e-cigarette for 1 week, and in good health. Exclusion criteria included allergies to e-liquid ingredients, current/past significant illness, current use of prescription medication (excluding contraception), uncorrected visual/hearing problems, loss of smell/taste, and, if female, pregnancy or breast feeding. Criteria were assessed by self-report except smoking status (cotinine detection ≥100 ng/mL) and pregnancy, which was verified by urine tests. In line with our ethics approval, a pregnancy test was not required if a female participant confirmed without doubt that she was not pregnant.

### Procedure

#### Screening and consent (20 min)

Participants were phone-screened to confirm eligibility and then provided written informed consent (Qualtrics). Participants were randomised and study packages were posted to participants (cotinine test, e-cigarette, charger, user manual, e-liquids, alcohol wipes and instructions). Women received a pregnancy test (except for one participant who reported being in a same-sex relationship and confirmed without doubt that she was not pregnant).

#### Session 1 (day 1, 20 min)

Participants completed the urine tests. They then showed their results to the researcher via a video call in order to be enrolled. Participants completed a Qualtrics survey assessing baseline measures (cigarette dependence, previous use of e-cigarettes, motivation to quit smoking and cigarette craving).

#### Study week (days 1–7)

Participants used their e-cigarette at least once per day and were instructed to abstain from smoking. Every evening, participants received a link to a 5 min Qualtrics survey via text message (e-cigarette use, cigarette craving, cigarette use and food cravings). A researcher checked data completion on days 3, 5 and 8, and participants were prompted if there were missing data.

#### Session 2 (day 8, 30 min)

Via Qualtrics, participants confirmed daily e-cigarette use, inputted puff count and duration of use, and reported the number of e-liquid bottles used. They completed the Questionnaire of Smoking Urges-Brief (QSU-Brief) before and after the cue exposure task. Secondary measures (enjoyment of e-cigarette, ease of transitioning, intentions to continue using an e-cigarette, intentions to quit smoking and motivation to quit smoking) were completed. Participants were no longer required to use their e-cigarette, and continued use was their choice.

#### Follow-up (day 15, 10 min)

Participants completed a phone interview reporting return to smoking, cigarettes per day and continuation of e-cigarette use (past week and future). They were debriefed (verbally and via email) and reimbursed £100. We planned to replace participants who withdrew, who fully relapsed to smoking, and those who failed to use the e-cigarette, complete the survey on ≥3 consecutive days, or complete session 2 within 48 hours.

### Materials and measures

#### E-cigarette and e-liquids

Participants received an Arc 5 tank-style e-cigarette (set to 12 watts) (https://www.totallywicked-eliquid.co.uk/arc-5) and Red Label freebase nicotine e-liquids (eg, 50:50 propylene glycol/vegetable glycerine; https://www.totallywicked-eliquid.co.uk/unflavoured-red-label). Participants in the flavoured condition selected two of four flavours based on a verbal list (blackcurrant, strawberry, vanilla, caramel). They were unable to sample flavours as the study was remote. E-liquid ingredients are provided in online supplemental table S1. Some people can detect some sweetness in Red Label unflavoured e-liquid (https://www.totallywicked-eliquid.co.uk/unflavoured-red-label). Puff count (quantity of power button presses) and duration of e-cigarette use (total time in seconds) were passively measured on the e-cigarette.

10.1136/tobaccocontrol-2021-056769.supp1 Supplementary data

E-liquid quantity and nicotine concentration were determined by reported cigarettes per day (online supplemental table S2). We surveyed ex-smoking current e-cigarette users (n=93) to identify average daily use and nicotine concentration to determine the supply for the current study (online supplemental table S3). To avoid compromising the study (eg, rationing, postal delays), we erred towards the upper end of supply (12, 16 or 20 10 mL bottles). After monitoring use, this was decreased (10, 14 or 18 10 mL bottles). Participants who smoked 5–19 or ≥20 cigarettes per day received an e-liquid nicotine strength of 1.0% (10 mg/mL) or 1.8% (18 mg/mL), respectively. This 1.0% (10 mg/mL) represents the average nicotine strength of purchased e-liquids,^39^ and the higher dose accommodated heavier smokers.

#### Questionnaires

We measured general cigarette craving daily using one item, ‘I craved a cigarette today’, from 0 ‘strongly disagree’ to 7 ‘strongly agree’. The mean average cigarette craving and peak cigarette craving (highest daily score) were derived. A single item was used to reduce participant burden. Single-item craving questions have face validity, although they cannot capture the diversity of craving experiences.^40^ We measured baseline and cue-elicited cigarette craving (post-task minus pre-task change scores) using the QSU-Brief.^41^

Secondary outcome measures were smoking lapse occurrence (return to smoking but recovered abstinence for ≥24 hours; no/yes), enjoyment of the e-cigarette (1 ‘not at all’ to 5 ‘a great deal’), ease of transitioning from smoking to using an e-cigarette (1 ‘very difficult’ to 5 ‘very easy’), intentions to continue using an e-cigarette and intentions to quit smoking (1 ‘definitely not’ to 5 ‘definitely’), and motivation to quit smoking (Readiness to Quit Ladder)^42^ after the study week, and return to smoking (no/yes) and continuation of e-cigarette use (past week and future; no/yes) at 1-week follow-up. Exploratory outcomes were sweet and savoury food cravings using a single item: ‘I craved sweet/savoury foods today’ (1 ‘strongly disagree’ to 7 ‘strongly agree’). Gender, age, ethnicity, employment status during the study week, cigarettes per day, duration of daily smoking and previous e-cigarette use described the sample. We assessed cigarette dependence using the Fagerström Test for Cigarette Dependence.^43 44^ Full measurement details can be found in the data dictionary.^45^

#### Cue exposure task

Phase 1 involved a computer-based presentation of 20 smoking-related images and 20 associated attention check questions. In phase 2, audio instructions asked participants to imagine situations in which they would normally smoke, while handling a cigarette box (or tobacco pouch), cigarette (or roll-up) and lighter. To check attention, participants entered two letters stated during the audio.

### Statistical analyses

We aimed to recruit 84 participants, which would provide 90% power at an alpha level of 0.005 to detect an effect size of Cohen’s *d*=0.92 and 80% power at an alpha level of 0.05 to detect an effect size of Cohen’s *d*=0.62. Cohen’s *d*=0.62 represents a mean difference between conditions of ~7 points on the QSU-Brief,^41^ which ranges from 10 to 70, and Cohen’s *d*=0.92 represents a mean difference of ~11 points. Reduction in QSU-Brief craving scores is associated with smoking cessation success. For example, patients who quit smoking by the 12th week of a smoking cessation intervention consistently reported QSU-Brief scores that were ~10 points lower than those who failed to quit smoking.^46^

Analyses were conducted using Stata (SE V.15). We used linear and logistic regression for continuous and binary outcomes, respectively. E-liquid flavouring was dummy-coded (0 unflavoured, 1 flavoured). Unadjusted analyses were compared with analyses adjusted for key baseline characteristics (age, gender, cigarette dependence, cigarettes per day and quit motivation). These were selected a priori based on potential associations with cigarette craving to increase the precision of estimates. We planned to adjust for puff count and duration of use but realised that this was inappropriate as these were not collected at baseline and therefore could not be used to improve the precision of estimates. As a sensitivity analysis, primary models were re-estimated when removing participants who experienced a smoking lapse and those who failed the attention checks (<100% performance). Finally, we explored the effects of e-liquid flavouring on food cravings, and changes in cigarettes per day, quit motivation and cigarette craving from baseline to study completion.

## Results

Study data, analysis code and associated documents are available at the University of Bristol data repository.^45^

### Participant characteristics

One hundred participants were screened, but 13 were ineligible, 2 withdrew and 1 was later replaced as their data were compromised (online supplemental figure S1). Participants (N=84, 55% male) were aged between 18 and 59 years. At baseline, cigarettes per day ranged from 5 to 30, duration of daily smoking ranged from <1 to 35 years, and 40% of participants had used an e-cigarette previously. Cigarette dependence ranged from 0 to 9, quit motivation ranged from 1 to 9, cigarette craving ranged from 14 to 52, and 55% of participants had not experienced a change in their employment status/environment. E-liquid flavour choices were as follows: blackcurrant, n=22; strawberry, n=23; vanilla, n=23; and caramel, n=16. Of the participants, 92% received the lower strength e-liquid (10 mg/mL) and 52% received 10 10 mL bottles of e-liquid. Usage ranged from 0.5 to 7 bottles. Participant baseline characteristics and e-cigarette usage during the study across conditions were similar, apart from duration of use, which was almost double in the flavoured condition (table 1).

**Table 1:** Participant baseline characteristics and e-cigarette usage

	Whole sample	Unflavoured condition	Flavoured condition
Demographics			
Age, years, mean (SD)	28.8 (9.9)	26.1 (7.7)	31.5 (11.2)
Gender, male, n (%)	46 (55)	26 (62)	20 (48)
Ethnicity, white, n (%)	76 (90)	37 (88)	39 (93)
Employment, working environment as normal, n (%)	46 (55)	21 (50)	25 (60)
Cigarette and e-cigarette use (baseline)			
Ever e-cigarette use, yes, n (%)	34 (40)	18 (43)	16 (38)
Cigarettes per day, mean (SD)	11.9 (4.8)	11.1 (5.1)	12.6 (4.4)
Duration of daily smoking, years, mean (SD)	9.3 (8.2)	7.5 (6.6)	11.1 (9.3)
Cigarette dependence, mean (SD)	3.4 (2.1)	3.2 (2.2)	3.5 (2.0)
Quit motivation, mean (SD)	5.5 (1.6)	5.3 (1.7)	5.6 (1.5)
Cigarette craving, mean (SD)	31.7 (9.0)	32.4 (9.0)	31.1 (9.1)
E-cigarette usage (after study week)			
E-liquid strength, 10 mg/mL, n (%)	77 (92)	38 (90)	39 (93)
E-liquid quantity used (bottles), mean (SD)	2.4 (1.5)	2.0 (1.3)	2.7 (1.7)
Puff count, mean (SD)	1434 (1219)	1333 (881)	1538 (1494)
Duration of use, in seconds, mean (SD)	4705 (8168)	3289 (1833)	6049 (11166)

N=84 for all variables except ‘puff count’ and ‘duration of use’ (measured passively on the device) due to missing or erroneous data (n=81 and n=76, respectively).

Cigarette dependence, quit motivation and cigarette craving were assessed using the Fagerström Test for Cigarette Dependence, Readiness to Quit Ladder and the Questionnaire of Smoking Urges-Brief, respectively.

e-cigarette, electronic cigarette.

### Primary outcomes

There was no clear evidence of an effect of e-liquid flavouring on average cigarette craving (mean difference 0.18, 95% CI −0.44 to 0.79, p=0.57, Cohen’s *d*=0.13) (unflavoured: M=4.6, SD=1.3; flavoured: M=4.8, SD=1.6), peak cigarette craving (mean difference −0.12, 95% CI −0.59 to 0.35, p=0.62, Cohen’s *d*=0.11) (unflavoured: M=6.2, SD=0.8; flavoured: M=6.1, SD=1.3) or cue-elicited cigarette craving (mean difference −0.21, 95% CI −3.86 to 3.43, p=0.91, Cohen’s *d*=0.03) (unflavoured: M=7.4, SD=9.3; flavoured: M=7.2, SD=7.4). The results did not differ after adjustment for baseline characteristics (table 2; see online supplemental table S4 for raw data). The results remained unchanged when excluding participants who experienced a smoking lapse (n=13, 15%) and those who failed the audio (n=1, 1%) and image (n=38, 45%; maximum 4/20 incorrect) attention checks (online supplemental table S5).

**Table 2:** Effect of e-liquid flavouring on all outcomes

Linear regressions (continuous outcomes)				
Outcome	Unadjusted		Adjusted	
	*b* (95% CI)	P value	*b* (95% CI)	P value
Cigarette craving (average)	0.18 (−0.44 to 0.79)	0.568	0.18 (−0.45 to 0.81)	0.573
Cigarette craving (peak)	−0.12 (−0.59 to 0.35)	0.616	−0.02 (−0.50 to 0.46)	0.934
Cue-elicited cigarette craving	−0.21 (−3.86 to 3.43)	0.907	0.78 (−3.04 to 4.60)	0.685
Enjoyment of e-cigarette	0.33 (−0.07 to 0.74)	0.107	0.30 (−0.14 to 0.74)	0.177
Ease of transitioning to using e-cigarette	−0.19 (−0.66 to 0.28)	0.422	−0.20 (−0.69 to 0.29)	0.423
Intentions to continue using e-cigarette	−0.17 (−0.60 to 0.27)	0.446	−0.38 (−0.80 to −0.05)	0.085
Intentions to quit smoking	0.21 (−0.28 to 0.71)	0.393	−0.05 (−0.48 to 0.39)	0.830
Motivation to quit smoking	0.12 (−0.74 to 0.98)	0.784	−0.30 (−1.07 to 0.48)	0.449
Sweet food craving (average)	0.40 (−0.13 to 0.94)	–	0.37 (−0.18 to 0.92)	–
Sweet food craving (peak)	0.57 (−0.01 to 1.15)	–	0.66 (0.05 to 1.27)	–
Savoury food craving (average)	−0.06 (−0.57 to 0.45)	–	−0.03 (−0.58 to 0.52)	–
Savoury food craving (peak)	−0.10 (−0.55 to 0.36)	–	−0.05 (−0.54 to 0.43)	–
**Logistic regressions (binary outcomes)**				
**Outcome**	**Unadjusted**		**Adjusted**	
	**OR (95% CI**)	**P value**	**OR (95% CI**)	**P value**
Smoking lapse occurrence	0.83 (0.25 to 2.73)	0.763	1.01 (0.29 to 3.55)	0.985
Return to smoking	0.70 (0.27 to 1.83)	0.464	1.19 (0.40 to 3.59)	0.755
Continuation of use (past week)	1.54 (0.24 to 9.71)	0.647	1.15 (0.15 to 8.64)	0.895
Continuation of use (future)	4.32 (0.46 to 40.35)	0.200	3.32 (0.31 to 35.70)	0.321

N=84. Condition code: unflavoured=0, flavoured=1. Unstandardised *b* coefficients.

Adjusted: adjusted for age, gender, cigarettes per day, cigarette dependence and quit motivation (baseline characteristics).

P values are not reported for exploratory analyses as (by definition) there was no hypothesis testing.

Primary measures: cigarette craving (average): mean average daily cigarette craving score (0 ‘strongly disagree’ to 7 ‘strongly agree’); cigarette craving (peak): highest daily cigarette craving score; cue-elicited cigarette craving: Questionnaire of Smoking Urges-Brief change scores (post-task minus pre-task).

Secondary measures: enjoyment of e-cigarette (1 ‘not at all’ to 5 ‘a great deal’), ease of transitioning to using e-cigarette (1 ‘very difficult’ to 5 ‘very easy’), intentions to continue using e-cigarette and intentions to quit smoking (1 ‘definitely not’ to 5 ‘definitely’), motivation to quit smoking (Readiness to Quit Ladder), and sweet and savoury food cravings (1 ‘strongly disagree’ to 7 ‘strongly agree’).

Binary outcomes were scored as 0=no and 1=yes.

e-cigarette, electronic cigarette.

### Secondary outcomes

There was no clear evidence of an effect of e-liquid flavouring on enjoyment of the e-cigarette, ease of transitioning from smoking to using an e-cigarette, intentions to continue using an e-cigarette, and intentions and motivation to quit smoking (table 2). There was no clear evidence of a difference in smoking lapse occurrence, or return to smoking and continuation of e-cigarette use at follow-up. The results did not differ after adjustment for baseline characteristics. Despite no difference between conditions, 94% of participants continued to use their e-cigarette and reported planning to use one in the future, while 27% continued to abstain from smoking.

### Exploratory outcomes

There was largely no clear evidence of an effect of e-liquid flavouring on food cravings (table 2). However, there was some evidence for peak sweet food craving (adjusted *b* 0.66, 95% CI 0.05 to 1.27, Cohen’s *d*=0.47) (unflavoured: M=4.9, SD=1.6; flavoured: M=5.5, SD=1.0). Compared with baseline, at follow-up, mean cigarettes per day had dropped by 9 (M=2.7, SD=3.8). Compared with baseline, at session 2, mean cigarette craving had decreased by 3 points (M=29.3, SD=11.9) and mean quit motivation had increased by 1.5 points (M=6.9, SD=2.0).

### Unplanned post-hoc analyses

We conducted Bayesian independent samples t-tests using JASP (V.0.14.1). This provided a Bayes factor 1 of 6.4 for average cigarette craving, 2.9 for peak cigarette craving and 4.0 for cue-elicited cigarette craving, which means that the data are approximately six, three and four times more likely to occur under the null hypothesis than the alternative hypothesis, respectively.^47^ Furthermore, there was no clear evidence of an effect of e-liquid flavouring on cigarettes per day (online supplemental information).

## Discussion

Contrary to our hypotheses, average, peak and cue-elicited cigarette craving did not appear to differ between participants using an e-cigarette containing unflavoured versus flavoured e-liquid for 1 week. We did not find evidence to indicate an effect of e-liquid flavouring on smoking lapse occurrence (during the study week), enjoyment of the e-cigarette, ease of transitioning from smoking to using an e-cigarette, intentions to continue using an e-cigarette, intentions to quit smoking, motivation to quit smoking (after study week), and return to smoking and continuation of e-cigarette use (1-week follow-up). These findings suggest that, during an initial switch from smoking to using e-cigarettes, there may be little impact of a fruit/sweet-flavoured e-liquid restriction on cigarette craving (if smokers choose to use unflavoured products). This is particularly interesting given the low population preference for (4%)^17^ and low interest in trying (5%)^37^ unflavoured e-liquids, and reports suggesting some e-cigarette users would stop using e-cigarettes or return to smoking if flavours were unavailable.^2^ However, we have only explored one potential mechanism by which flavours may play a role in smoking reduction and cessation.

Exploratory analyses suggested that e-liquid flavouring might have a medium-sized effect (based on conventions described by Cohen^48^) on peak sweet food craving, with scores 9% higher in the flavoured condition (equating to a difference of 0.6 points on a 7-point scale). Some e-cigarette users report using sweet-flavoured e-liquids for appetite control and weight loss,^49^ and to replace eating sweet foods,^50^ whereas others avoid these flavours, citing concerns about weight gain.^51^

Our study makes a unique contribution and has some important strengths compared with the few experimental studies that have investigated the effects of e-cigarette flavouring on cigarette craving to date.^29 30^ First, we included an unflavoured condition, which is important as unflavoured e-liquids tend to be exempt from policies that restrict e-cigarette products. Second, the vaping period was 1 week (vs minutes) long, and participants could use their e-cigarette ad libitum at home (vs a laboratory). This meant we observed behaviour more naturally, and our findings have enhanced ecological validity. Third, participants in the flavoured condition were given some choice of flavours and nicotine concentration was tailored to their need. Fourth, we used a tank-style e-cigarette, which is commonly used,^2^ generally considered to be satisfying and reduces cigarette cravings,^26^ increasing the generalisability of our results. However, the nicotine delivery profile of the Arc 5 e-cigarette is unknown. Fifth, we included a follow-up assessment to explore continuation of use. Finally, there was no attrition during the study.

Our study has limitations and additional questions must be addressed before attempting to inform policy. First, we cannot rule out the possibility of a smaller but nevertheless meaningful effect that our study was underpowered to detect. The upper bound of the 95% CI for cue-elicited craving is ~4 points on the QSU-Brief, which could still be important at a population level. Second, we did not include a tobacco flavour condition due to funding and time restraints. Future studies could include this third condition because tobacco flavours are seldom included in bans, and cigarette craving may be alleviated more by flavours that have existing conditioned associations with smoking.^30^ Indeed, some participants anecdotally reported enjoying the unflavoured e-cigarette because it resembled smoking (ie, tasted like a cigarette/tobacco). We are unaware of supporting research evidence, although some vaping blogs have corroborated this.^52^ Third, this study examined short-term e-cigarette use among smokers not currently attempting to quit and with proxy outcomes for smoking behaviour. It is important to determine if differences emerge over longer time periods and during smoking quit attempts, where the primary outcomes are smoking reduction and cessation. Tobacco flavours may be important during the initial transition from smoking to e-cigarette use (or dual use), and then flavour variety (non-tobacco) may support e-cigarette engagement and smoking relapse prevention (eg, by reducing sensory-specific satiety). For example, adult dual users report a preference for tobacco flavours at initiation of e-cigarette use, while non-tobacco flavours are more common among former smokers.^11^ Furthermore, e-cigarette users who use sweet-flavoured (vs tobacco or unflavoured) products are more likely to stop smoking.^53^ Fourth, we did not biologically verify smoking abstinence. As remote testing was initiated in response to COVID-19, we did not have funding to assess expired carbon monoxide remotely. Fifth, 40% of our sample were not naïve to e-cigarette use and prior experiences may have influenced craving. However, we decided to include previous e-cigarette users to make our sample more representative of the population of interest^2^ and to avoid hampering recruitment. Sixth, although we conducted pilot research to determine typical e-liquid use based on cigarettes per day and we provided participants with an ample supply, some participants may have been underdosed, which could have affected cigarette cravings. However, due to randomisation, we do not expect systematic variation between experimental conditions. Seventh, we aggregated daily craving scores across time, which may have reduced statistical power. Finally, outcomes were measured after smokers were exposed to unflavoured e-liquid. While our findings are promising for those who have already switched from combustible to e-cigarettes, questions remain regarding whether market restrictions of non-tobacco flavoured e-liquids would impact smokers’ motivations to switch to e-cigarettes (and unflavoured e-liquids) in the first place, particularly among those who have not previously sampled unflavoured e-liquids.

## Conclusions

We found no clear evidence to suggest that e-liquid flavouring (fruit/sweet vs unflavoured) has an effect on cigarette craving, experiences of e-cigarette use, and smoking cessation intentions and motivations after 1 week of use. Policy changes regarding the restriction of e-liquid flavours need to be carefully considered in light of the evidence. These findings may be negated if smokers are not motivated to try unflavoured products and initiate use, or if flavours play a role in adherence to e-cigarette use for smoking reduction and cessation. Further research is needed to establish if differences emerge over longer periods of exposure, among smokers attempting to quit, and whether there are any effects on smoking cessation outcomes.

**What this paper adds**

- Several countries have implemented policies restricting flavoured e-cigarette products, although the impact on smokers is unknown.
- Non-tobacco flavoured e-liquids appear to be important for smoking cessation, but how and why are currently unclear.
- Here, smokers randomised to use an e-cigarette with flavoured (fruit/sweet) or unflavoured nicotine-containing e-liquids for 1 week did not differ in cigarette craving, experiences of using e-cigarettes or smoking cessation intentions/motivations.
- Further research is needed to establish if differences emerge over longer periods of exposure and whether there is any effect on smoking cessation.
- These findings suggest that, during an initial switch from smoking to using e-cigarettes, there may be little impact of using unflavoured e-liquids on cigarette craving if fruit/sweet-flavoured e-liquids are restricted.

## Data Availability

Data are available in a public, open access repository. Study data, analysis code and associated documents are available at the University of Bristol data repository (https://data.bris.ac.uk/data/dataset/2keqznqqitdqs24ahc1e0b2x6o).
