# Supplementary material for: Effects of electronic cigarette e-liquid flavouring on cigarette craving
Source: Tob Control. 2021 Nov 17;32(e1):e3–9. doi: 10.1136/tobaccocontrol-2021-056769 (PMC7614335; doi:10.1136/tobaccocontrol-2021-056769)
Supplement: Supplementary data [file tobaccocontrol-2021-056769supp001.pdf]

E-LIQUID FLAVOURING AND CIGARETTE CRAVING

Supplementary Information

Supplementary Table S1. Constituents of the e-liquids.

Flavour	Ingredient list
Blackcurrant	Propylene Glycol, Vegetable Glycerine, Water, Nicotine, Vanillin, Raspberry Ketone, Ethyl Acetate, Ethyl Butyrate, Acetic Acid
Strawberry	Propylene Glycol, Vegetable Glycerine, Water, Nicotine, Ethyl Acetoacetate, 2 Methyl Butyric Acid, Ethyl Butyrate, Cis-3-Hexenol
Vanilla	Propylene Glycol, Vegetable Glycerine, Water, Nicotine, Vanillin
Caramel	Propylene Glycol, Vegetable Glycerine, Water, Nicotine, Vanillin, Butyric Acid
Unflavoured	Propylene Glycol, Vegetable Glycerine, Water, Nicotine

Dyer ML, *et al. Tob Control* 2023; 32:e3–e9. doi: 10.1136/tobaccocontrol-2021-056769

Supplementary Table S2. E-liquid quantities and dose provided for participants.

Reported cigarettes per day	Number of 10ml bottles for low dose (1.0%; 10mg/ml)	Number of 10ml bottles for high dose (1.8%; 18mg/ml)
**At the start of the study (from 01/07/2020)**		
5-14	12 (120 ml, 15 ml per day)	
15-19	16 (160 ml, 20 ml per day)	
20-24		16 (160 ml, 20 ml per day)
25+		20 (200 ml, 25 ml per day)
**After monitoring participant use (from 31/07/2020)**		
5-14	10 (120 ml, 15 ml per day)	
15-19	14 (160 ml, 20 ml per day)	
20-24		14 (160 ml, 20 ml per day)
25+		18 (200 ml, 25 ml per day)

Note: We initially provided participants with 12, 16 or 20 10ml bottles of e-liquid, depending on their reported cigarettes per day before the study. After monitoring use for the first month of data collection, this was decreased to 10, 14, or 18 10ml bottles (depending on reported cigarettes per day), because we learned that we were oversupplying participants to a higher degree than necessary. Participants in the flavoured condition received 50% of the total bottles in each of their chosen two flavours.

Supplementary Table S3. Results of the pilot survey with ex-smoking current e-cigarette users to determine e-liquid supply.

Reported cigarettes per day (previously)	Number of bottles we will send (total ml, nicotine %)	Reported ml/day: N (%)			Based on survey, approx. N (%) who will be over- or undersupplied	
		≤10	11-20	21+	Oversupplied	Correct amount or undersupplied
5-14	12 (120 ml, 15 ml/day, 1.0%)	15 (88%)	1 (6%)	1 (6%)	15 (88%)	2 (12%)
15-19	16 (160 ml, 20 ml/day, 1.0%)	9 (60%)	5 (33%)	1 (7%)	9 (60%)	6 (40%)
20-24	16 (160 ml, 20 ml/day, 1.8%)	17 (63%)	9 (33%)	1 (4%)	17 (63%)	10 (37%)
25+	20 (200 ml, 25 ml/day, 1.8%)	13 (52%)	7 (28%)	5 (20%)	20 (80%)	5 (20%)

Note: N = 84. Table excludes survey respondents who reported smoking 1-4 cigarettes per day (n = 4) and ‘other’ (n = 5).

We conducted a survey of ex-smokers who were currently vapers (n=93) to identify how much e-liquid that they use daily, at which dosage and how much they used to smoke. As expected, vaping amount and nicotine dosage increased with previous cigarettes per day, which justifies our graded increase with smoking heaviness. The table shows the amount we planned to send, survey outcomes and estimated proportion with potential surplus. E-liquid use was highly variable, and we erred towards upper end of estimates (rather than using the average for example) as we needed to measure natural behaviour. We did not want participants to ration their vaping for example if e-liquid supply was running low.

Dyer ML, *et al. Tob Control* 2023; 32:e3–e9. doi: 10.1136/tobaccocontrol-2021-056769

Supplementary Figure S1. CONSORT flow diagram.

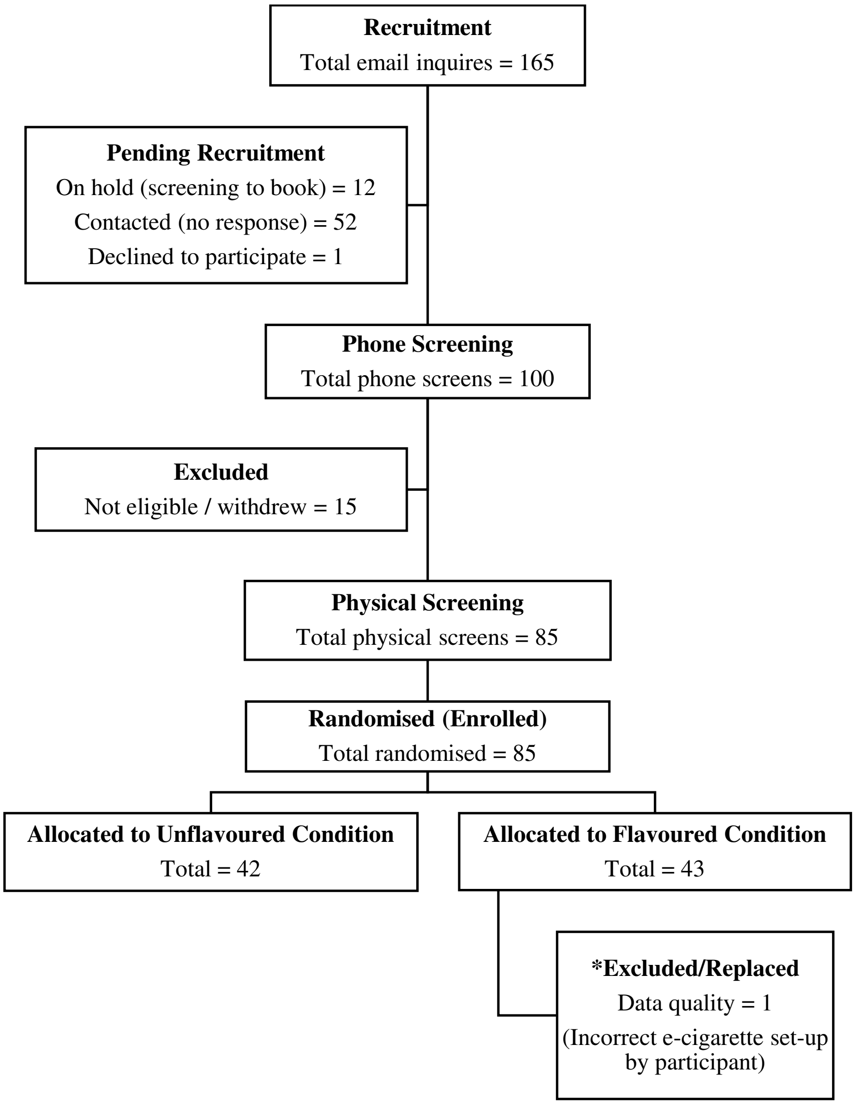

*One additional participant was randomised to the flavoured condition to replace a participant who completed the study, but whose data was unusable. The final sample therefore consisted of 42 participants in each flavour condition.

Supplementary Table S4. Raw outcome data, by flavour condition.

Outcome	Unflavoured	Flavoured
	**Mean (Standard Deviation)**	
Cigarette craving (average)	4.6 (1.3)	4.8 (1.6)
Cigarette craving (peak)	6.2 (0.8)	6.1 (1.3)
Cue-elicited cigarette craving	7.4 (9.3)	7.2 (7.4)
Enjoyment of e-cigarette	3.1 (0.9)	3.4 (0.9)
Ease of transitioning to using e-cigarette	3.4 (1.0)	3.2 (1.1)
Intentions to continue using e-cigarette	4.0 (1.1)	3.8 (0.9)
Intentions to quit smoking	3.5 (1.1)	3.7 (1.2)
Motivation to quit smoking	6.9 (2.0)	7.0 (1.9)
Sweet food craving (average)	3.4 (1.4)	3.8 (1.1)
Sweet food craving (peak)	4.9 (1.6)	5.5 (1.0)
Savoury food craving (average)	4.3 (1.3)	4.2 (1.0)
Savoury food craving (peak)	5.7 (1.1)	5.6 (0.9)
	**'Yes' N (%)**	
Smoking lapse occurrence	7 (16.7)	6 (14.3)
Return to smoking	32 (76.2)	29 (69.1)
Continuation of use (past week)	39 (92.9)	40 (95.2)
Continuation of use (future)	38 (90.5)	41 (97.6)

Note: N = 84. Condition code: unflavoured = 0, flavoured = 1. Primary measures: Cigarette craving (average) = mean average daily cigarette craving score (0 'strongly disagree' to 7 'strongly agree'). Cigarette craving (peak) = highest daily cigarette craving score. Cue-elicited cigarette craving = Questionnaire of Smoking Urges-Brief change scores (post- minus pre-task). Secondary measures: enjoyment of e-cigarette (1 'not at all' to 5 'a great deal'), ease of transitioning to using e-cigarette (1 'very difficult' to 5 'very easy'), intentions to continue using e-cigarette and intentions to quit smoking (1 'definitely not' to 5 'definitely'), motivation to quit smoking (Readiness to Quit Ladder), and sweet and savoury food cravings (1 'strongly disagree' to 7 'strongly agree').

Supplementary Table S5. Effect of e-liquid flavouring on primary outcomes (if no smoking lapse and no attention check failures).

	Unadjusted			Adjusted		
**Outcome**	***b* (95% CI)**	***p***	**N**	***b* (95% CI)**	***p***	**N**
**Cigarette craving (average)**						
No lapse	0.12 (-0.55 to 0.80)	.716	71	0.09 (-0.64 to 0.83)	.802	71
Audio check passed	0.17 (-0.45 to 0.79)	.589	83	0.17 (-0.46 to 0.81)	.590	83
Image check passed	-0.10 (-1.03 to 0.83)	.830	46	-0.03 (-0.94 to 0.89)	.952	46
**Cigarette craving (peak)**						
No lapse	-0.14 (-0.68 to 0.39)	.594	71	0.01 (-0.56 to 0.58)	.968	71
Audio check passed	-0.14 (-0.61 to 0.33)	.557	83	-0.04 (-0.51 to 0.44)	.881	83
Image check passed	-0.35 (-1.08 to 0.38)	.342	46	-0.32 (-1.01 to 0.38)	.365	46
**Cue-elicited cigarette craving**						
No lapse	-0.79 (-4.73 to 3.14)	.689	71	1.21 (-3.06 to 5.48)	.574	71
Audio check passed	-0.33 (-4.01 to 3.35)	.858	83	0.62 (-3.19 to 4.42)	.748	83
Image check passed	1.70 (-3.86 to 7.27)	.540	46	1.84 (-4.19 to 7.88)	.540	46

Condition code: unflavoured = 0, flavoured = 1. Unstandardised b coefficients. Adjusted = adjusted for age, gender, cigarettes per day, cigarette dependence, quit motivation (baseline characteristics). Cigarette craving (average) = mean average daily cigarette craving score (0 ‘strongly disagree’ to 7 ‘strongly agree’). Cigarette craving (peak) = highest daily cigarette craving score. Cue-elicited cigarette craving = Questionnaire of Smoking Urges-Brief change scores (post- minus pre-task).

Unplanned Post-hoc Analyses

Unplanned exploratory analyses also showed that there was no clear evidence of an effect of e-liquid flavouring on cigarettes per day during the study week (mean difference 0.03, 95% CI -0.30 to 0.35, $p = .87$), or at one week follow up (mean difference -0.05, 95% CI -1.72 to 1.62, $p = .96$).
